# Radiographic evaluation of patellofemoral proportional mismatch in small-breed dogs with medial patellar luxation: Implications for stifle joint morphology and surgical planning

**DOI:** 10.14202/vetworld.2026.2293-2303

**Published:** 2026-06-05

**Authors:** Ekkapol Akaraphutiporn, Khemupsorn Kaewmahing, Pichamon Chunhasewee, Sansinee Fuengsil, Tawanrat Chaiyaphan, Chalika Wangdee

**Affiliations:** 1 Department of Veterinary Surgery, Faculty of Veterinary Science, Chulalongkorn University, Bangkok, Thailand; 2 Center of Excellence in Biomaterial Engineering in Medical and Health, Chulalongkorn University, Bangkok, Thailand

**Keywords:** anatomical measurement, medial patellar luxation, morphometric analysis, patellofemoral mismatch, radiographic evaluation, skyline radiograph, small-breed dogs, trochlear dysplasia

## Abstract

**Background and Aim:**

Medial patellar luxation (MPL) is a common orthopedic disorder in small-breed dogs and is closely associated with abnormalities of the patellofemoral joint. Although trochlear dysplasia is widely recognized, the proportional relationship between the patella and distal femur remains insufficiently explored. This study aimed to perform a comprehensive radiographic morphometric evaluation of patellofemoral structures to identify anatomical factors associated with MPL and to assess their relevance for surgical planning.

**Materials and Methods:**

A total of 69 stifles from 46 small-breed dogs were evaluated and categorized into control, MPL grade II, and MPL grade III groups. Standardized ventrodorsal, mediolateral, and skyline radiographic projections were used to measure patellar width (PW), patellar height (PH), patellar length (PL), femoral width (FW), femoral condyle size (FC), trochlear width (TW), trochlear depth (TD), trochlear length (TL), and sulcus angle (SA). Morphometric ratios, including PW/TW, PH/TD, PL/TL, PW/FW, and PH/FC, were calculated. Statistical analysis was conducted using one-way analysis of variance with Tukey’s post hoc test (p < 0.05).

**Results:**

Patellar dimensions (PW, PH, PL) did not differ significantly among groups, indicating that patellar morphology alone was not associated with MPL severity. In contrast, distal femoral parameters showed significant alterations. The MPL grade III group exhibited significantly reduced TD compared with grade II, and increased SA compared with both control and grade II groups, reflecting progressive trochlear flattening. The PH/TD ratio was significantly higher in the MPL grade III group, suggesting a proportional mismatch between patellar size and trochlear groove depth. Other ratios did not show significant differences, although PL/TL demonstrated an increasing trend in affected stifles.

**Conclusion:**

MPL in small-breed dogs is primarily associated with distal femoral morphological abnormalities rather than absolute patellar size. Trochlear shallowness and increased SA, along with an elevated PH/TD ratio, highlight the importance of patellofemoral proportional mismatch in disease pathogenesis. These findings emphasize the need for comprehensive morphometric evaluation during preoperative planning to optimize surgical outcomes.

## INTRODUCTION

The patella plays a critical role in stifle joint biomechanics. Embedded within the tendon of the quadriceps femoris muscle, it functions as a sesamoid bone that enhances the mechanical efficiency of quadriceps contraction during stifle extension. Its smooth articular surface and curvature allow coordinated movement within the femoral trochlear groove, helping to maintain joint stability throughout flexion and extension. Proper congruency between the patella and the trochlear groove is therefore essential because any disruption of this relationship alters joint mechanics and predisposes the stifle to dysfunction [1, 2].

Patellar luxation is one of the most common orthopedic conditions in dogs and involves displacement of the patella from its normal position within the femoral trochlear groove. Although luxation can be medial, lateral, or bidirectional, medial patellar luxation (MPL) predominates and occurs far more frequently in small and toy breeds, reportedly up to 12 times more often than in large dogs [3]. Breeds such as Chihuahuas, Pomeranians, Yorkshire Terriers, and Poodles are particularly predisposed [4–7]. Females are affected more frequently, and bilateral involvement is reported in 20% to 52% of cases [3, 7, 8].

The pathogenesis of MPL is multifactorial and reflects abnormalities not only within the stifle but throughout the quadriceps extensor mechanism, which includes the quadriceps muscle, patella, patellar ligament, femoral trochlea, and tibial tuberosity [2, 9]. Developmental misalignment of this mechanism is widely accepted as a key etiologic factor, and proximal skeletal deformities, such as coxa vara, may contribute to medial deviation. A shallow femoral trochlear groove is a well-recognized feature of MPL, although whether this represents a primary malformation or a developmental consequence of abnormal patellar tracking remains debated [6, 10, 11]. Radiographic, computed tomographic, and ultrasonographic studies consistently demonstrate decreased trochlear depth (TD) and increased sulcus angle (SA) in affected dogs, with these changes becoming more pronounced as luxation severity increases [12–16].

Similar observations have been documented in human medicine, where trochlear dysplasia is a major contributor to patellar instability. Quantitative imaging demonstrates wider SA and shallower TD in affected patients [17–19]. More recent studies have also highlighted the role of patellar morphology itself, showing that smaller patellae or a reduced medial facet width significantly increase the risk of recurrent instability [20]. These findings emphasize the principle that patellofemoral congruence is fundamental to joint stability across species.

Despite recognition of the importance of trochlear morphology in MPL pathogenesis, relatively little attention has been given to the influence of patellar size, shape, and proportion relative to the distal femur in small-breed dogs. Most veterinary studies have emphasized femoral conformation, potentially overlooking the contribution of patellar morphology to stifle biomechanics. A more comprehensive understanding of patellofemoral morphometry may improve diagnostic precision and strengthen preoperative planning. Identifying specific morphologic patterns associated with MPL offers a clinically derivable framework that bridges the practicality of standard radiographs with the anatomical insights typically reserved for more advanced imaging modalities.

Current veterinary literature predominantly focuses on femoral abnormalities, particularly trochlear dysplasia, as the principal factor contributing to MPL, while comparatively limited attention has been directed toward the proportional relationship between the patella and distal femur. Although reductions in TD and increases in SA have been consistently reported, the role of patellar morphology in relation to trochlear geometry remains insufficiently characterized in small-breed dogs. Furthermore, most existing studies rely on absolute linear measurements rather than proportional indices that may better reflect functional congruency within the patellofemoral joint. This limitation restricts the ability to fully interpret biomechanical interactions and may lead to incomplete preoperative assessment. In addition, the clinical utility of radiographically derived ratios, such as PH/TD, as indicators of patellofemoral mismatch has not been comprehensively evaluated in naturally occurring MPL cases. Therefore, a critical gap exists in understanding how proportional morphometric relationships contribute to disease development and severity.

The objective of this study was to perform a detailed radiographic morphometric analysis of the stifle in small-breed dogs using standardized projections to evaluate both patellar and distal femoral structures. Specifically, this study aimed to compare morphometric parameters and derived ratios between dogs with and without MPL to identify anatomical variables associated with disease development. It was hypothesized that dogs with MPL would exhibit a shallower TD and a wider SA compared with controls. In addition, the PH/TD ratio was proposed as a clinically relevant indicator of patellofemoral mismatch that may provide additional insight beyond absolute measurements and support improved surgical planning.

## MATERIALS AND METHODS

### Ethical approval

The study protocol was reviewed and approved by the Institutional Animal Care and Use Committee of the Chulalongkorn University Laboratory Animal Center, Bangkok, Thailand, under Animal Use Protocol No. 2231050. All procedures involving animals were performed in accordance with the ethical principles and institutional guidelines governing the use of animals in research and clinical investigations.

Written informed consent was obtained from all dog owners before enrolment in the study. Owners were informed about the objectives of the study, radiographic procedures, anesthetic management, and the intended use of clinical data. Participation was voluntary, and confidentiality of patient information was maintained throughout the study.

The study included only client-owned dogs presented to the Surgical Unit of the Small Animal Hospital, Faculty of Veterinary Science, Chulalongkorn University, for routine orthopedic examination and diagnostic evaluation. No experimental induction of disease, invasive experimental intervention, or unnecessary manipulation was performed. All diagnostic procedures were integrated into standard clinical management and performed solely for the benefit of patient evaluation and treatment planning.

Radiographic examinations were conducted under general anesthesia using individualized anesthetic protocols tailored to each dog’s physical condition based on the American Society of Anesthesiologists classification. Continuous monitoring was performed throughout anesthesia to ensure animal safety and welfare. Appropriate positioning and gentle handling techniques were applied during radiographic acquisition to minimize stress, discomfort, and the risk of injury.

All efforts were made to reduce pain, distress, and procedural risk during clinical examination and imaging. Dogs were managed according to accepted veterinary standards of care, and all procedures complied with national and institutional regulations related to animal ethics, welfare, and biomedical research. The study adhered to internationally accepted ethical standards for veterinary clinical research involving privately owned animals.

### Study period and location

This study was conducted from October 2022 to September 2023 at the Surgical Unit of the Small Animal Hospital, Faculty of Veterinary Science, Chulalongkorn University, Bangkok, Thailand. Case enrolment and data collection were carried out over the defined study period during which eligible small-breed dogs presenting with or without MPL were prospectively evaluated under standardized clinical and radiographic conditions.

### Study design

This was a prospective observational study designed to evaluate radiographic morphometric parameters of the stifle joint in small-breed dogs. Dogs presented to the Surgical Unit were screened for eligibility based on clinical and orthopedic examination findings. Data collected included breed, sex, age, body weight, and relevant orthopedic conditions. Dogs younger than 1 year of age, those with a history of previous MPL surgery, and those diagnosed with other orthopedic disorders resulting in hindlimb deformities were excluded. Eligible dogs were categorized into three groups: control, MPL grade II, and MPL grade III.

Grading of MPL was performed according to criteria adapted from the Singleton grading system [21]. Briefly, grade I was defined as patellar luxation that could be manually induced but returned spontaneously to the trochlear groove, with minimal tibial deviation; grade II was characterized by frequent patellar luxation that could be manually reduced but readily reluxated, often with mild tibial rotation and intermittent limb carrying; grade III was defined as permanent patellar luxation that could be manually reduced, accompanied by moderate tibial rotation and a shallow or flattened trochlear groove; and grade IV was characterized by permanent, non-reducible patellar luxation with severe tibial rotation and an absent or convex trochlear groove.

### Radiographic positioning

Three standardized views were obtained for each stifle: ventrodorsal, mediolateral, and skyline. All radiographs were acquired with side markers and a metallic calibration ball measuring 25 mm in diameter to ensure consistency in image scaling and orientation.

**Figure 1:**
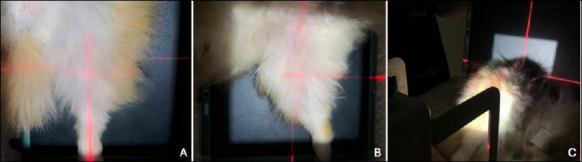
Clinical photographs illustrating the positioning of a Pomeranian for stifle radiography: (A) ventrodorsal, (B) mediolateral, and (C) skyline projections. Side markers and the metallic calibration ball were excluded to provide a clearer view of limb orientation and primary beam centering.

For the ventrodorsal view, dogs were positioned in dorsal recumbency with the hips and stifles extended, and the stifle joint placed as close as possible to the radiographic cassette. A vertical beam was centered over the patella. Proper alignment criteria included centering the patella within the femoral trochlear groove, bisecting the fabellae by their respective femoral cortices, maintaining parallel alignment of the femurs with the long axis of the pelvis, extending the hips in neutral rotation, and ensuring visibility of the tip of the lesser trochanter on the medial femur (Figure 1A).

For the mediolateral view, dogs were positioned in lateral recumbency with the limb to be examined closest to the cassette. A vertical beam was centered over the femoral epicondyles, with the pelvic limb positioned at its normal standing joint angles. Acceptable projection criteria required superimposition of the medial and lateral femoral condyles (Figure 1B).

For the skyline view, dogs were positioned in dorsal recumbency with the pelvic limb flexed to 90°. The radiographic cassette was placed on the abdomen, cranial to the stifle joints, and securely held in position. A horizontal beam was directed at the patella, with collimation adjusted to include both the patella and femoral condyles (Figure 1C) [22].

### Radiographic measurements

Based on the three radiographic views obtained, nine morphometric parameters were assessed: patellar width (PW), patellar height (PH), patellar length (PL), femoral width (FW), femoral condyle size (FC), trochlear width (TW), TD, trochlear length (TL), and SA. Prior to measurement, each radiographic image was calibrated using the metallic calibration ball to ensure accuracy and consistency of size scaling.

All measurements were performed independently by four researchers using computer-based image analysis software (vPOP-Pro version 2.9.2; VetSOS Education Ltd., Wales, UK), with all observers blinded to group allocation. Inter-observer reliability was evaluated using the intraclass correlation coefficient, with values greater than 0.80 considered acceptable. For parameters that could be assessed using more than one view, specifically PW, PH, and PL, the mean value from both views was calculated and used as the final measurement for each dog.

**Figure 2:**
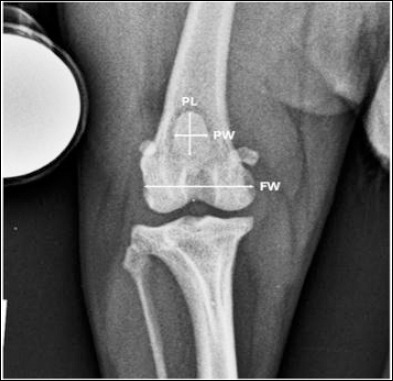
Measurements in the ventrodorsal view showing patellar width (PW), patellar length (PL), and femoral width (FW).

In the ventrodorsal view (Figure 2), PW, PL, and FW were measured. PW was defined as the widest horizontal dimension of the patella, while PL was measured as the longest vertical dimension of the patella in this projection. FW was measured by drawing a line perpendicular to the long axis of the femur at the level of the base of the intercondylar fossa, extending from the medial to the lateral FC, and representing the transverse width of the distal femur at this level.

**Figure 3:**
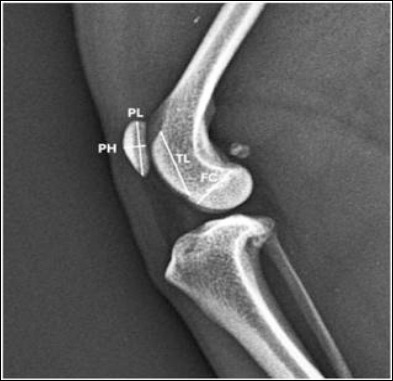
Measurements in the mediolateral view showing patellar height (PH), patellar length (PL), femoral condyle size (FC), and trochlear length (TL).

In the mediolateral view (Figure 3), PH, PL, FC, and TL were measured. PH was recorded as the maximum vertical dimension of the patella, while PL was again defined as the longest dimension of the patella. FC was measured as the linear distance from the origin of the long digital extensor muscle to the caudal femoral cortex, following Blumensaat’s line, which serves as a radiographic landmark for the intercondylar roof [23]. TL was defined as the distance from the proximal extent of the femoral trochlear ridge to Blumensaat’s line, representing the longitudinal dimension of the trochlear groove in the sagittal plane [24].

**Figure 4:**
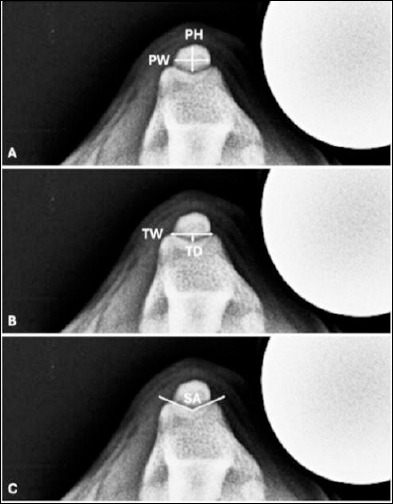
Measurements in the skyline view: (A) patellar width (PW) and patellar height (PH), (B) trochlear width (TW) and trochlear depth (TD), and (C) sulcus angle (SA).

In the skyline view (Figure 4), measurements included PW, PH, TW, TD, and SA. In this projection, PW and PH were determined as the widest and highest dimensions of the patella, respectively. TW was measured by drawing a line connecting the highest points of the medial and lateral femoral condyles, representing the width of the trochlear groove at its opening [25]. TD was measured as the perpendicular distance from this line to the deepest point of the sulcus [16]. Finally, SA was defined as the angle formed at the deepest point of the intercondylar groove by two lines extending from the highest points of the medial and lateral femoral condyles, reflecting the angular configuration of the femoral sulcus [11].

### Radiographic analysis

All raw measurements obtained independently by the four observers were averaged to calculate a mean value for each morphometric parameter, thereby minimizing interobserver variability and improving measure-ment reliability. Both patellar and distal femoral measurements were recorded in millimeters (mm). In addition to absolute measurements, specific ratios were calculated to assess proportional relationships between patellar and distal femoral structures. These included patella–trochlear and patella–condyle ratios, calculated using mean parameter values. The following ratios were evaluated: PW/TW, PH/TD, PL/TL, PW/FW, and PH/FC.

### Statistical analysis

Data are presented as mean ± standard deviation. The chi-square test was used to assess homogeneity of sample demographics among study groups. One-way analysis of variance, followed by Tukey’s multiple comparison test, was used to compare morphometric parameters and calculated ratios between groups. The 95% confidence intervals were calculated where appropriate to estimate the precision of the measurements. A p-value of < 0.05 was considered statistically significant. All statistical analyses were performed using GraphPad Prism software (GraphPad Software Inc., La Jolla, CA, USA).

## RESULTS

### Demographic characteristics of study population

In total, 69 stifles from 46 small-breed dogs were evaluated (Table 1), comprising 33 Chihuahua stifles (48%) and 36 Pomeranian stifles (52%). The stifles were categorized into three groups: 8 stifles (12%) in the control group, 47 stifles (68%) in the MPL grade II group, and 14 stifles (20%) in the MPL grade III group. The mean age of all dogs was 47.8 ± 36.6 months. Dogs in the control group were statistically older than those in both the MPL grade II (p = 0.002) and MPL grade III groups (p = 0.003), with approximately double the mean age. The mean body weight was 3.4 ± 1.1 kg, and no significant differences were observed among groups with respect to body weight, breed distribution, sex, or affected limb.

**Table 1:** Demographic characteristics of dogs in the control group and those with MPL grades II and III.

Characteristics	Control	MPL grade II	MPL grade III	p-value
Number of stifles	8	47	14	
Age (months)	88.8 ± 48.6ᵃ^,^ᵇ	41.9 ± 32.6ᵃ	44.2 ± 28.2ᵇ	0.002
Body weight (kg)	3.15 ± 1.12	3.52 ± 1.11	3.29 ± 1.15	0.625
Breed				0.233
Chihuahua	6	20	7	
Pomeranian	2	27	7	
Sex				0.109
Male	6	21	4	
Female	2	26	10	
Limb side				0.673
Right	3	24	8	
Left	5	23	6	

MPL = medial patellar luxation. Data are presented as mean ± standard deviation. a = Significant difference between control and MPL grade II groups, b = Significant difference between control and MPL grade III groups.

### Patellar morphology

With respect to patellar morphology, PW, PH, and PL did not differ significantly among the control, MPL grade II, and MPL grade III groups (Table 2). Mean patellar dimensions across all stifles were as follows: PW 5.16 ± 0.68 mm, PH 3.26 ± 0.41 mm, and PL 8.36 ± 0.85 mm. These findings indicate that patellar size and shape were not associated with MPL severity in this population.

**Table 2:** Morphometric measurements of the patella and distal femur in control stifles and stifles with MPL grades II and III.

Variables	Control	MPL grade II	MPL grade III	p-value
Patellar morphology (mm)				
PW	5.22 ± 0.93	5.18 ± 0.67	5.06 ± 0.58	0.810
PH	3.22 ± 0.48	3.29 ± 0.39	3.18 ± 0.45	0.668
PL	8.39 ± 1.34	8.37 ± 0.84	8.32 ± 0.54	0.976
Distal femur morphology (mm)				
TW	6.06 ± 0.79	5.78 ± 0.72	5.77 ± 0.97	0.641
TD	0.78 ± 0.13	0.79 ± 0.21ᶜ	0.60 ± 0.21ᶜ	0.013
TL	13.92 ± 1.93	12.78 ± 1.21	12.66 ± 1.55	0.082
FW	15.92 ± 2.04	15.38 ± 1.73	15.26 ± 0.94	0.622
FC	7.30 ± 1.37	7.44 ± 1.05	6.88 ± 0.65	0.198
SA (degrees)	150.3 ± 3.3ᵇ	149.5 ± 7.2ᶜ	156.8 ± 6.4ᵇ^,^ᶜ	0.003

MPL = medial patellar luxation, PW = patellar width, PH = patellar height, PL = patellar length, TW = trochlear width, TD = trochlear depth, TL = trochlear length, FW = femoral width, FC = femoral condyle size, SA = sulcus angle. Data are presented as mean ± standard deviation. b = Significant difference between control and MPL grade III groups, c = Significant difference between MPL grade II and MPL grade III groups

### Distal femoral morphology

By contrast, significant differences were identified in trochlear morphology (Table 2). TD was significantly shallower in the MPL grade III group than in the MPL grade II group (p = 0.049, 95% confidence interval 0.001–0.375). In addition, the SA was significantly greater in the MPL grade III group than in both the control (p = 0.036, 95% confidence interval 0.4–12.6) and MPL grade II groups (p = 0.016, 95% confidence interval 1.5–13.1). These results demonstrate progressive flattening of the trochlear groove with increasing MPL severity.

### Patella–trochlear ratios

Patella–trochlear ratios, including PW/TW, PH/TD, and PL/TL, were evaluated to characterize the relationship between patellar dimensions and distal femoral morphology (Table 3). The PH/TD ratio was markedly higher in MPL grade III stifles, measuring approximately 1.6 times the control value and 1.4 times that of MPL grade II. A statistically significant difference was observed between the MPL grade III and control groups (p = 0.012, 95% confidence interval 0.5–3.5). Although not statistically significant, PL/TL demonstrated a trend toward increasing values in MPL-affected stifles.

**Table 3:** Morphometric ratios of the patella and distal femur in control stifles and stifles with MPL grades II and III.

Variables	Control	MPL grade II	MPL grade III	p-value
Patella–trochlear ratios				
PW/TW	0.87 ± 0.09	0.89 ± 0.11	0.89 ± 0.11	0.898
PH/TD	3.89 ± 0.58ᵇ	4.39 ± 1.34	6.04 ± 3.43ᵇ	0.011
PL/TL	0.61 ± 0.02	0.67 ± 0.07	0.67 ± 0.07	0.063
Patella–condyle ratios				
PW/FW	0.33 ± 0.03	0.33 ± 0.03	0.33 ± 0.02	0.954
PH/FC	0.47 ± 0.05	0.45 ± 0.05	0.47 ± 0.03	0.142

MPL = medial patellar luxation, PW = patellar width, PH = patellar height, PL = patellar length, TW = trochlear width, TD = trochlear depth, TL = trochlear length, FW = femoral width, FC = femoral condyle size. Data are presented as mean ± standard deviation. b = Significant difference between control and MPL grade III groups.

### Patella–condyle ratios

Patella–condyle ratios, including PW/FW and PH/FC, showed no significant differences among groups (Table 3).

### Overview of patellofemoral morphology in MPL

The present study examined patellar and distal femoral morphology in small-breed dogs with and without MPL, focusing on Chihuahuas and Pomeranians. These breeds represent a large proportion of MPL diagnoses and therefore serve as an important model for understanding anatomic risk factors. Although trochlear dysplasia, particularly a shallow femoral trochlear groove and reduced medial trochlear ridge height, is widely recognized as the primary anatomical abnormality associated with MPL, relatively few studies have investigated whether patellar morphology itself, or its relationship to distal femoral structures, also contributes to patellar maltracking. To address this gap, we evaluated both patellar and distal femoral dimensions using standard orthogonal radiographs (ventrodorsal and mediolateral), supplemented by a skyline projection. Although the skyline view is not routinely obtained in clinical MPL assessment, its inclusion allowed improved visualization of trochlear groove depth, SA, and patellar position. The use of three radiographic projections provided a more comprehensive anatomical assessment and offered an approximation of near three-dimensional evaluation, helping to characterize structural relationships that may not be apparent on routine orthogonal views.

### Patellar morphology and its limited role in MPL

With respect to patellar morphology, our results demonstrated no significant differences in PW, PH, or PL among control dogs and those with MPL grades II and III. Because the study population consisted exclusively of small-breed dogs with similar body weights, patellar size was unlikely to be influenced by differences in overall skeletal size. These findings suggest that patellar dimensions alone are not a major determinant of MPL severity in these breeds. Contrary to previous reports, our study found no significant differences in PH, PW, or PL among the three groups. Earlier literature has suggested that patellar thickness may be reduced in MPL-affected dogs, proposing that patellar hypoplasia may contribute to instability [26, 27].

Discrepancies between our findings and previous studies may be explained by differences in measurement methodology. Whereas some earlier studies relied solely on the skyline projection to measure patellar thickness, we incorporated both mediolateral and skyline views and used averaged values to improve consistency. This methodological difference may account for variations reported across studies. It is also important to recognize that the patella is a three-dimensional, almond-shaped structure, and the linear measurements used in this study may not fully capture variations in patellar contour, volume, articular surface geometry, or facet development. Such features may influence patellofemoral congruence and stability but cannot be reliably assessed using two-dimensional radiography. Further investigation using computed tomography or three-dimensional surface reconstruction may provide deeper insight into whether subtle patellar morphological variations contribute to MPL pathogenesis [28].

### Clinical relevance of patellar height

The PH in our study was consistently around 3 mm across all groups, which aligns with previously published values for both normal and MPL-affected small-breed dogs [16]. This consistency suggests that absolute PH is relatively uniform among these breeds and is unlikely to be a major determinant of MPL. However, this value may be useful when estimating the amount of recession required during trochlear groove deepening procedures in small-breed dogs [29, 30].

### Distal femoral morphology and trochlear dysplasia

Among the distal femoral parameters evaluated, TD and SA differed significantly between groups. These findings are consistent with well-established associations between trochlear shallowness and MPL [11, 15]. The increasing SA observed in MPL-affected dogs, particularly those with grade III luxation, supports the presence of trochlear dysplasia and parallels findings in human medicine, where widened SA (>145°) are recognized indicators of patellar instability [14]. Although the SA values reported in our study differ slightly from those in previous veterinary literature, such variation is likely attributable to differences in radiographic positioning during skyline imaging [14, 16].

Trochlear groove depth is not uniform along its length; therefore, the degree of stifle flexion determines which segment of the groove is visualized. Small deviations in flexion angle can substantially influence SA and TD measurements. Standardizing the stifle angle using a goniometer and confirming positioning with a mediolateral horizontal beam view may improve measurement reliability. Alternatively, computed tomography scanning offers a more accurate assessment of the entire trochlear groove and eliminates projection-related variability [12, 31].

### Patella–trochlear and patella–condyle relationships

To minimize the confounding effects of body size and to better characterize anatomical relationships, we evaluated patella–trochlear and patella–condyle ratios. The patella–trochlear ratios included PW/TW, PH/TD, and PL/TL, which represent patellar dimensions relative to the trochlear groove, essentially the functional “seat” in which the patella resides. By contrast, the patella–condyle ratios included PW/FW and PH/FC, reflecting the relative development of the patella and femoral condyles in corresponding dimensions.

Most ratios did not differ significantly between groups, with the exception of the PH/TD ratio. This finding aligns with earlier results demonstrating differences in TD and SA among groups. Although the significant difference in PH/TD was observed only between the control group and the MPL grade III group, it supports the association between trochlear shallowness and more severe MPL. These observations suggest that while trochlear shallowness is commonly associated with MPL, its severity and consistency become more evident in higher-grade cases [11, 26]. Although the PL/TL ratio did not reach statistical significance, it demonstrated an increasing trend in MPL-affected stifles, suggesting that TL may also be insufficient relative to patellar size [24]. This observation highlights a potential mismatch in longitudinal patellofemoral congruity, which could contribute to instability even when absolute patellar dimensions are within normal limits.

### Implications for surgical planning

These findings support the rationale for surgical procedures aimed at improving patellofemoral congruence, such as trochleoplasty or sulcoplasty. While traditionally focused on increasing absolute groove depth, our radiographic analysis identifies the PH/TD ratio as a clinically derivable indicator of patellofemoral incongruity. This index complements absolute trochlear parameters to guide tailored surgical corrections; for instance, a high PH/TD ratio highlights a proportional mismatch that may require more significant recession to achieve stability. Furthermore, our data suggest that restoring adequate groove length may also be important [6, 32]. Ensuring appropriate trochlear geometry in all dimensions, including depth, width, and length, may improve patellar tracking and postoperative stability. Intraoperative evaluation of groove width and length may therefore be beneficial in preventing persistent maltracking.

### Study limitations

This study has several limitations. The relatively small sample size, particularly in the control group, may have reduced statistical power and limited subgroup comparisons. MPL grades I and IV were excluded because of low case numbers, restricting representation of the full disease spectrum. Furthermore, the focus on Chihuahuas and Pomeranians from a single hospital population may introduce selection bias, and these findings may not fully generalize to all small breeds. Variability in stifle flexion angles during skyline radiograph acquisition may also have influenced certain measurements; standardizing flexion angles using a goniometer would likely reduce this variability. Finally, conventional radiography cannot fully capture three-dimensional joint geometry. Advanced imaging modalities such as computed tomography or magnetic resonance imaging would provide a more complete evaluation of patellar and distal femoral morphology and may help identify subtle shape features that contribute to MPL.

## CONCLUSION

This study provides a comprehensive radiographic morphometric evaluation of the patellofemoral joint in small-breed dogs and demonstrates that MPL is primarily associated with distal femoral morphological alterations rather than absolute patellar size. Specifically, TD was significantly reduced and SA was significantly increased in higher-grade MPL, indicating progressive trochlear flattening. Among the evaluated indices, the PH/TD ratio emerged as the most clinically relevant parameter, reflecting a proportional mismatch between patellar dimensions and trochlear groove depth. In contrast, PW, PH, and PL did not differ significantly among groups, suggesting that patellar morphology alone does not substantially influence disease severity in this population. Additionally, the observed trend toward increased PL/TL highlights a potential contribution of longitudinal incongruity to patellar instability.

From a clinical perspective, these findings emphasize that surgical decision-making for MPL should extend beyond absolute measurements of TD to include proportional relationships within the patellofemoral joint. The PH/TD ratio, in particular, offers a practical and radiographically accessible indicator of incongruity that may guide the extent of trochlear recession during corrective procedures. Furthermore, consideration of TL, in addition to depth and width, may improve restoration of patellar tracking and enhance postoperative stability. These insights support a more individualized and geometry-based approach to surgical planning.

A major strength of this study lies in the use of standardized multi-view radiographic assessment, including the skyline projection, which enabled improved visualization of trochlear morphology and patellar alignment. The evaluation of both absolute measurements and proportional ratios provides a more comprehensive understanding of patellofemoral biomechanics. In addition, the focus on commonly affected small-breed dogs enhances the clinical relevance of the findings for routine veterinary practice.

Future research should incorporate advanced imaging modalities such as computed tomography or magnetic resonance imaging to enable detailed three-dimensional characterization of joint geometry and to validate radiographic indices identified in this study. Larger, multicenter studies including a broader range of breeds and all MPL grades are warranted to improve generalizability. Longitudinal investigations assessing postoperative outcomes in relation to preoperative morphometric parameters would further clarify the predictive value of ratios such as PH/TD. Moreover, integration of biomechanical modeling may help to better understand the functional implications of patellofemoral mismatch.

In conclusion, MPL in small-breed dogs is strongly associated with trochlear dysplasia and patellofemoral proportional mismatch rather than isolated patellar size abnormalities. The identification of the PH/TD ratio as a key indicator of incongruity underscores the importance of incorporating proportional morphometric assessment into clinical evaluation and surgical planning. These findings contribute to a more refined understanding of MPL pathogenesis and support the development of targeted, anatomically informed treatment strategies aimed at improving long-term joint stability and clinical outcomes.

## DATA AVAILABILITY

The data generated during the study are included in the manuscript.

## AUTHORS’ CONTRIBUTIONS

EA and CW: Study design and data interpretation. EA: Statistical analysis and manuscript writing. KK, PC, SF, and TC: Study execution and data collection. CW: Supervision of the study. All authors have read, reviewed, and approved the final version of the manuscript.
